# Effect of Organic Acid Addition Before Fermentation on the Physicochemical and Sensory Properties of Cherry Wine

**DOI:** 10.3390/foods13233902

**Published:** 2024-12-03

**Authors:** Wenbo Yang, Zhenzhen Lv, Hui Liu, Qiang Zhang, Chengkui Qiao, Muhammad Nawaz, Zhonggao Jiao, Jiechao Liu

**Affiliations:** 1 Zhengzhou Fruit Research Institute, Chinese Academy of Agricultural Science, Zhengzhou 450009, China; yangwenbo@caas.cn (W.Y.); liuhui@caas.cn (H.L.);; 2 Zhongyuan Research Center, Chinese Academy of Agricultural Science, Xinxiang 453000, China

**Keywords:** cherry wine, malic acid, lactic acid, antioxidant activity, non-volatile compounds, volatile organic compounds, color, sensory evaluation

## Abstract

Lack of acidity is the main reason for the spoilage of cherry wine, and for insufficient aroma and mouthfeel. In this study, the initial acidity of cherry purees was adjusted to 3.50, 4.15, 4.80 and 5.45 g/kg by using malic acid, lactic acid and a mixture of the two before fermentation. And the effects of different organic acid additions on the physicochemical profiles and sensory properties of cherry wines were investigated. Our findings suggest that organic acid addition can inhibit the formation of volatile acid and enhance ethanol production, while having a negative effect on their polyphenol contents. These additions can be utilized as carbon sources during cherry wine fermentation and affect its metabolism. Among them, the application of malic acid with lactic acid was shown to have more metabolically active effects on non-volatile compounds, and enhanced the total volatile organic compounds by 14.04%–66.92%. MC-4.80 and MLC-4.80 had the highest total VOC content and odor score in the sensory evaluation. However, the addition of large amounts of acids reduced the acidity score and overall acceptability of cherry wine. In conclusion, adjusting the initial acid content to 4.15 g/kg before fermentation significantly improved the quality of cherry wines, and the combination of malic acid and lactic acid was more effective for cherry winemaking. This finding evidenced that organic acid addition could be an effective strategy for improving the quality of cherry wines.

## 1. Introduction

The Chinese cherry [*Cerasus pseudocerasus* (Lindl.) G. Don], belonging to the genus *Cerasus* of the Rosaceae family, has been cultivated for over 3000 years in China [1,2]. Due to its delicious taste and high nutritional and ornamental value, the Chinese cherry has been consumed widely in recent years, and has been playing a vital role in the promotion of the local economy with the rise in farm tourism [3]. However, Chinese cherries are difficult to preserve because of their thin skin and high water content; they are often eaten directly after picking or processed into juices and wines [4]. The consumption of cherry wine has become popular in China due to its high content of health-promoting bioactive compounds, such as polyphenols, anthocyanins, vitamins and melatonin [5].

However, the low acidity of cherries (pH > 3.7) is an urgent challenge during wine production, because it increases the incidence of microbial spoilage, and promotes the formation of biogenic amines and volatile acid [6]. Moreover, the insufficient acidity of the material cannot allow highly aromatic cherry wine to meet the preferences of customers, and affects other sensorial properties of cherry wine, such as appearance and taste [7,8,9]. Castaneda-Ovando et al. [10] suggested that the proportion of flavylium ions would increase in a highly acidic fermentation environment, which contributes to the red and purple color in red wine. The appropriate acidity can promote astringency, inhibit sweetness and confer freshness and vitality in fruit wine, which give a more balanced and softer mouthfeel [11,12,13,14]. In addition, Volschenk et al. [9] confirmed that high total acidity can positively contribute to the acid hydrolysis of non-volatile flavor compounds, thus promoting the release of a floral aroma and other flavor precursor content in fruit. And the changes in pH caused by acidic content in wine can also affect enzyme activity related to aroma metabolism, which results in a change in the aroma composition [15]. Therefore, proper acidification treatment provides the possibility of improving the quality of cherry wine, and also satisfies the growing consumer demand for highly acidic cherry wine.

Currently, the direct addition of organic acids, such as tartaric acid, citric acid, malic acid or lactic acid, is commonly used for wine acidification due to convenient operation, and usually performed before bottling [16,17]. However, the effects of organic acid addition before fermentation on wine quality are rarely reported. Organic acids are identified as the pivotal contributors to malolactic fermentation and involved in microbial metabolism during or after alcoholic fermentation, resulting in great changes in the composition of the final product [18]. Malolactic fermentation is known as the process responsible for flavor profiles, such as those created by alcohols, esters, aldehydes and volatile fatty acids [19,20]. Apart from this, they also provide carbon sources for lactobacillus growth and maintain the activities of microorganisms. Insufficient involvement of organic acids would limit the growth of organisms and the process of malolactic fermentation. Among the organic acids, malic acid is easily degraded by most wine microorganisms, and commonly used as an inducer for malolactic fermentation [21]. Lactic acid is the universal metabolite of malolactic fermentation, and also the main aroma precursor for esterification during winemaking [22]. Thus, it is hypothesized that the addition of malic acid or lactic acid could enhance the flavor of cherry wine.

In order to investigate whether these two acids could be utilized as substrates to promote aroma formation during fermentation and the appropriate initial acid concentration required for cherry wine fermentation, different dosages of malic acid, lactic acid and their mixture were used to adjust the acidity of cherry must before alcohol fermentation. And their effects were compared with a control group via estimating their physicochemical indexes and non-volatile and volatile profiles. A sensory evaluation was performed to identify satisfaction with different cherry wine samples. This work can provide an effective strategy for dealing with the problems of less fruity aromas and disharmony in the taste of cherry wine, and provide guidance for low-acidity fruit brewing.

## 2. Materials and Methods

### 2.1. Materials and Chemicals

Fresh cherries (*Chinese cherry*) were picked at commercial maturity in Zhenping county, Henan province, China, in 2023. The initial cherry composition was as follows: 11° Brix, titratable acidity 2.84 g/kg, pH 4.16. Commercial *S. bayanus* BV818 was purchased from Angel Yeast Co., Ltd. (Yichang, China).

The chemicals used in wine production were food grade additives. Sucrose (99.9%) was purchased from Guangxi Laibin Dongtang Fenghuang Co., Ltd. (Laibin, China). Lactic acid (≥60%) was purchased from Zhuangmei Biological Co., Ltd. (Hanchuan, China). L-malic acid (≥99.0%) was purchased from Changmao Biochemical Engineering Co., Ltd. (Changzhou, China). K_2_S_2_O_5_ (≥93%) was purchased from Zibo Changcheng Chemical Factory (Zibo, China). NaOH (95%), Na_2_CO_3_ (≥99.5%), NaNO_2_ (99%), Al (NO_3_)_3_, CuSO_4_ (99.0%) and K_4_FeC_6_N_6_·3H_2_O (99.0%) were obtained from Shanghai Macklin Biochemical Co. (Shanghai, China). NaCl (≥99.8%), C_4_H_4_O_6_KNa·4H_2_O (99.0%) and potassium hydrogen phthalate (99.99%) were purchased from Shanghai Guoyao Chemical Reagent Co., Ltd. (Shanghai, China). Folin–Ciocalteu’s phenol regent, gallic acid (CAS No. 149-917; ≥98.0%), catechin (CAS No. 154-23-4; ≥99.0%), 1,1-Diphenyl-2-picrylhydrazy (DPPH, CAS No. 1898-66-4; ≥90%), acetonitrile (CAS No. 75-05-8; ≥99.9%; LC-MS) and 2-octanol (CAS No. 74858; ≥99.5%, GC) were purchased from Sigma-Aldrich, Co. (St. Louis, MO, USA). Formic acid (CAS No. 64-18-6; ≥99.5%) was purchased from Dikma Technologies Inc. (Lake Forest, CA, USA).

### 2.2. Cherry Wine Fermentation

The cherry wine was produced according to the method reported by Yue et al. [23]. Cherries were destemmed and crushed, then supplemented with sucrose to reach a total reducing sugar concentration of 220 g/L. Three replicates of approximately 0.7 kg cherries per treatment were collected, and treated by addition of K_2_S_2_O_5_ (0.14 g/L, equivalent to 70 mg/L SO_2_). Then, malic acid (MC), lactic acid (LC) and malic–lactic acid (MLC: malic and lactic acid were mixed at 1:1) were added to adjust the titratable acid content to 3.50 g/kg, 4.15 g/kg, 4.80 g/kg and 5.45 g/kg, respectively. After that, the cherry puree was inoculated with *S. bayanus* BV818 (200 mg/L). Alcohol fermentation was completed in 1 L glass bottles maintained at 20 ± 2 °C until the sugar content was not changed significantly. Then, the cherry must was filtered and transferred to 500 mL bottles, K_2_S_2_O_5_ (0.04 g/L, equivalent to 20 mg/L SO_2_) was added and the mixture was stored at 20 °C for 30 days for aging, and the resulting cherry wines were stored at −20 °C before analysis.

### 2.3. General Physicochemical Composition

The determination of physicochemical parameters was carried out based on the National Standard of the People’s Republic of China GB/15038-2006 (General Administration of Quality Supervision, Inspection and Quarantine Standardization Administration of China, 2006) [24]. The residual sugar was determined with Fehling’s reagent, and the results were calculated using the glucose conversion coefficient. The titratable acidity (TA) and volatile acidity were determined by acid–base titration with 0.1 mol/L and 0.05 mol/L standard NaOH solution, and the results were calculated using the malic acid and acetic acid conversion coefficient. Alcohol content was determined with an alcohol meter and the dry extract was determined by the pycnometer method. The pH was determined using a digital pH meter (PHS-3C, Inesa, Shanghai, China) at room temperature (26 °C).

### 2.4. Color Analysis

Chromatic characteristics of cherry wine samples were determined by the CIELAB method. All samples were filtered through a 0.45 μm filter. The absorbance of samples was measured at 450, 520, 570 and 630 nm with an ultraviolet spectrophotometer (Specord 50, Analytic Jena, Jena, Germany) with reference to the ultrapure water (Academic, Milli-Q, Molsheim, France) and the path length was 2 mm. The color parameters ($L^{*}$, $a^{*}$, $b^{*},$ $C^{*}$, $h^{*}$ and ΔE) were calculated according to the formula proposed by Bai [17].

### 2.5. Total Phenolics, Total Flavonoids and Antioxidant Activity

Total phenol contents (TPCs) were determined by the Folin–Ciocalteu method [25] with modifications. In brief, 0.2 mL of samples or standard solutions was mixed with 2.5 mL Folin–Ciocalteu reagent. After incubation for 5 min at 50 °C (HCM 100-pro, Dargon, Beijing, China), 2 mL Na_2_CO_3_ (75 g/L) was added, and the mixture was kept for 30 min in the dark (26 °C); the absorbance was measured at 760 nm (Specord 50, Analytic Jena, Jean, Germany). The results were expressed as microgram of gallic acid equivalent per milliliter of cherry wine (μg GAE/mL).

Total flavonoid contents (TFCs) were determined by using a colorimetric assay [26]. Briefly, 1 mL of samples or standard solutions was mixed with 0.3 mL 5% NaNO_2_, and kept for 6 min at room temperature (26 °C); then, 0.3 mL 10% Al (NO_3_)_3_ was added to the mixture. After 6 min, 1.4 mL NaOH (1 mol/L) was added. The absorbance of the mixture was determined at 510 nm after 15 min against an appropriate blank. The TFC content was expressed as the catechin equivalent in μg CE/mL.

Antioxidant activity was measured as the DPPH radical scavenging capacity, following Zhao et al. [27], with some modifications. A total of 50 μL of each diluted wine was mixed with 200 μL DPPH solution (10 mmol/L). The absorbance of the mixture was determined at 517 nm (SpectraMax i3x, Molecular Devices, CA, USA) after it was held in the dark for 30 min. The relative antioxidant activity was calculated using the following equation:

(1) $$\%Inhibition=\left[\left(A_{DPPH}-A_{sample\;}\right)/A_{DPPH}\right]\times 100$$

where $A_{DPPH}$ is the absorbance of the DPPH solution without the sample, and $A_{sample}$ is the absorbance of the DPPH solution with the sample. The results were expressed as the % inhibition of DPPH radicals.

### 2.6. UPLC-Q-TOF/MS Analysis

Non-volatile compounds in cherry wine samples were analyzed according to Bi et al. [28], with slight modifications. The 10 µL samples were analyzed with a UPLC-Q-TOF/MS system, consisting of a Waters ACQUITY UPLC I-Class instrument (Waters, Milford, MA, USA) coupled with a Sciex Triple TOF 4600 quadrupole–time of flight mass spectrometer (AB Sciex, Foster City, CA, USA). A Waters ACQUITY UPLC HSS T3 column (100 mm × 2.1 mm× 1.8 µm) was used for the separation at 35 °C. The mobile phase and gradient profile are described in Table 1. All solvents and solutions were filtered with a 0.22 µm filter.

The Q-TOF/MS was operated in electrospray ionization (ESI) mode with positive and negative ions, and the parameters were as follows: the ion spray voltage for the positive mode and negative mode were 5500 V and −4500 V; the ion source gas 1 (GS1), ion source gas 2 (GS2) and curtain gas (CUR) were 55 psi, 60 psi and 35 psi, respectively; the temperature was 550 °C; and the decluttering potential was 100 V for positive mode and −100 V for negative mode. The full scan of the TOF masses ranged from 50 to 1200 Da for positive mode, and 100–1000 Da for negative mode. APCI positive/negative calibration solution was used for real time correction of the instrument’s mass accuracy.

The mass spectrometry data were analyzed with Peak View V2.2 (AB Sciex, CA, USA), and the compounds were identified by comparing the fragmentation patterns, fragment ions and isotope peak of the primary and secondary MS data with a self-established metabolite database and the Sciex public database: All in One and the TCM MS/MS library. The ionic chromatographic peak area of the identified metabolites was integrated with Multi Quant V.3.0.2 (AB Sciex, CA, USA) and was used to express the relative content for further statistical analysis.

### 2.7. E-Nose Analysis

E-nose analysis of cherry wine followed previous research with modifications [29]. The E-nose (PEN 3, Airsense Analytics, Schwerin, Germany) was equipped with 10 sensors, the performances of which are listed in Table 2. The electronic nose was warmed up for 10 s before sampling to achieve the operating temperature. A 2 mL 50-fold diluted sample was placed in a 15 mL headspace vessel and equilibrated at 26 °C for 10 min. The sensors were set as follows: the sample interval was 1 s, the flush time was 40 s, the measurement time was 60 s, the pre-sampling time was 5 s and the carrier gas velocity was 300 mL/min. The sensor intensity is defined as G/G0, where G0 and G are the resistance of the sensor in zero gas and sample gas, respectively. Each sample was analyzed at least six times, and the results were analyzed with Win-Muster software (Version 1.6.2.22, Airsense Analytics GmbH, Schwerin, Germany).

### 2.8. GC-MS Analysis

Volatile organic compounds (VOCs) were analyzed by HS-SPEM-GC-MS as described by Xiao et al. [30], with modifications. A total of 5 mL cherry wine, containing 2 g NaCl and 20 μL 2-octanol (internal standard, 413 μg/mL) was placed in a 15 mL headspace vial, and equilibrated for 30 min at 40 °C under an agitation speed of 500 r/min ( PC-420D, Corning, NY, USA). Before extraction, the SPME fiber (50/30 μm DVB/CAR/PDMS, SPME, Bellefonte, PA, USA) was conditioned at 250 °C for 40 min by being inserted into the injection port of GC-MS. Then, it was injected into the headspace vial for 30 min (40 °C) and inserted into the injection port immediately to desorb for 8 min at 250 °C under a splitless mode. An 8860-gas chromatograph (GC) coupled to a 5977-mass selective detector (MS) (Agilent, Santa Clara, CA, USA) was employed for separation and detection analyses. The DB-225 ms column (30 m × 0.25 mm × 0.25 μm, Agilent, USA) was used to perform the chromatographic separations. The oven temperature program is listed in Table 3. Helium was used as the column carrier gas with a constant flow rate of 1 mL/min. The transfer line, ion trap and quadrupole temperature were 280 °C, 230 °C and 150 °C. The MS parameters included electron impact ionization with electron energy of 70 eV at 1 s/scan, full scan mode and a mass range of m/z 50–550. A blank run was carried out to ensure no carryover of analytes from previous injections before sampling. The VOCs were identified by comparing their mass spectral data with the NIST 1.1 mass spectral database. The relative concentration of VOCs was calculated according to the following equation:

(2) $$Concentration\left(\frac{\mu g}{L}\right)=\frac{Peak\;of\;compound}{Peak\;of\;inernal\;standard}\times concentration\;of\;internal\;standard$$

### 2.9. Sensory Evaluation

Sensory evaluation was carried out following the method of Huang et al. [31], with some modification. The sensory scoring group consisted of twenty laboratory members, including ten women and ten men between 20 and 60 years old. Samples were divided into three groups according to the kinds of organic acid addition: the MC, MLC and LC groups. Each group of samples for evaluation was randomly placed into disposable plastic cups at 26 °C, which were rinsed with drinking water after each sensory evaluation. Testers scored the wines according to their color, aroma, acidity, clarity, mouthfeel and acceptability. A nine-point scale (1: extremely dislike; 5: neither like nor dislike; 9: extremely like) was used to score satisfaction. Then, the samples with high scores in each group were selected for sensory evaluation again according to the same method. The average score for each attribute was calculated and displayed in a spider diagram.

### 2.10. Statistical Analyses

Experiments were conducted three times, and the results were expressed as the mean ± standard deviation. Analysis of variance (ANOVA) was performed by using SPSS16.0 (2008), and Tukey’s multiple comparison was used to assess the significance of the treatment effects (*p* < 0.05). Orthogonal partial least squares–discriminant analysis (OPLS-DA) was processed using MetaboAnalyst 6.0. The relative concentrations of non-volatile and volatile compounds were normalized and visualized with TBtools V2.01 as a heatmap.

## 3. Results and Discussion

### 3.1. General Physicochemical Composition

**Figure 1:**
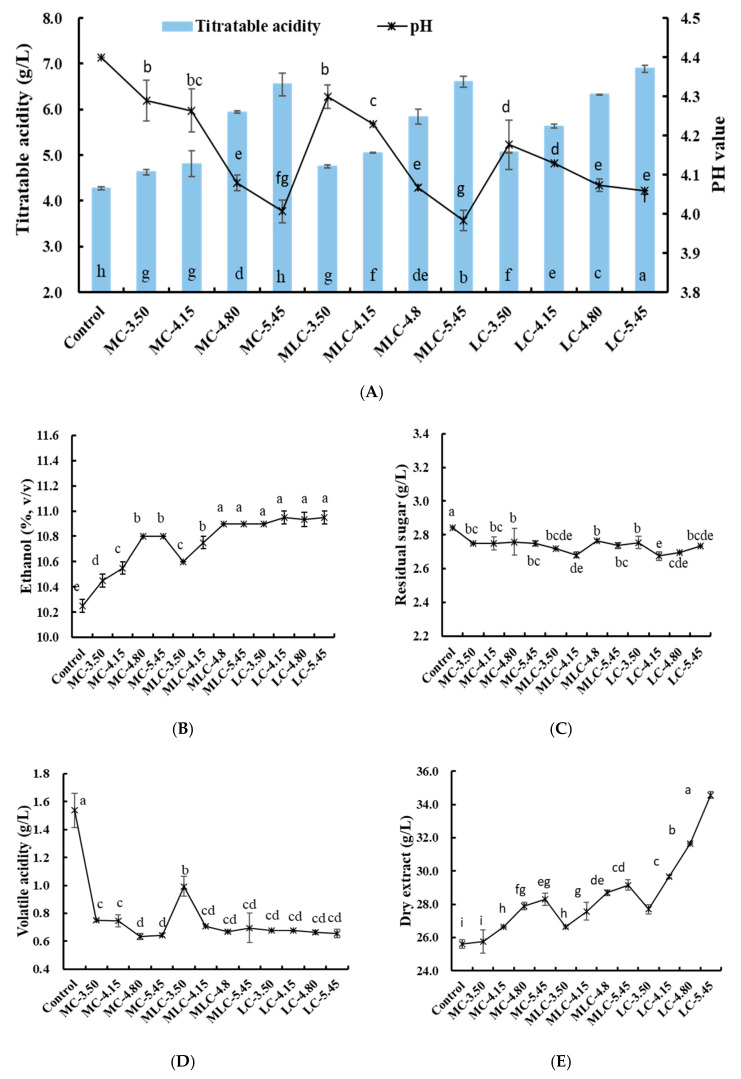
The physicochemical parameters of wine samples with different organic acid treatments. (**A**) pH and titratable acidity; (**B**) ethanol; (**C**) residual sugar; (**D**) volatile acidity; (**E**) dry extract. Difference lowercase letters represent significant difference (ANOVA, *p* < 0.05). (In the MC-3.50, MC-4.15, MC-4.80 and MC-5.45 groups, respectively, the initial acid contents of the cherry must were adjusted to 3.50, 4.15, 4.80 and 5.45 g/kg by using malic acid; in the MLC-3.50, MLC-4.15, MLC-4.80 and MLC-5.45 groups, they were adjusted by the mixture of malic acid and lactic acid; in the LC-3.50, LC-4.15, LC-4.80 and LC-5.45 groups, they were adjusted by lactic acid).

The effects of acid addition on the physicochemical properties of cherry wine are presented in Figure 1. In this study, the TAs of all experimental groups were significantly higher than that of the control group, increasing with the increased acid addition, accompanied by a pH level decrease as well. Among the groups, the LC group showed a higher TA than the other groups, while the changes in pH value were slight, decreasing from 4.14 to 4.06 when the initial TA increased from 3.50 to 5.45 g/kg. In contrast, the pH levels of MC and MLC varied widely, decreasing from 4.29 to 4.02 and 4.30 to 3.98, respectively. It is interesting that the MC and MLC groups showed higher pH levels when the initial TAs were 3.50 and 4.15 g/kg, while these levels became significantly lower than in the LC group as the initial acid concentration increased. The initial acid concentration and kinds of acid were probably the important factors affecting wine fermentation. The addition of malic acid leads to a more sufficient malolactic fermentation and is accompanied by the increase in pH level, which is caused by the conversion between C4 dicarboxylic malic acid and C3 monocarboxylic lactic acid [9]. However, the malolactic fermentation is limited by the excessive acids, and the excessive organic acid is the main reason for the pH decreasing, and the various organic acid profiles in cherry wine result in different pH values after fermentation [32].

In addition, the change in pH caused by acid addition could significantly affect other physicochemical parameters of wines [33]. In this research, the acidification did not affect the sugar consumption but significantly affected the ethanol content, volatile acidity and dry extract of wine. Organic acid addition significantly reduced the volatile acidity of cherry wines (0.63–0.99 g/L), and caused it to be less than the acceptable limit (1.2 g/L acetic acid equivalents) according to OIV [34]. Moreover, these groups also showed higher alcohol content and more dry extract than the control. During wine fermentation, the sugar was mainly utilized by yeast and converted into ethanol. The constant sugar consumption indicated that the addition of organic acid did not affect the activity of yeast. Volatile acid is basically derived from acetic acid, which is produced from ethanol by acetobacteria metabolism [35]. And the decrease in volatile acids and increase in ethanol in the acid treatment groups might be caused by the decrease in the initial pH value of cherry must, which could inhibit the growth of acetic acid bacteria [36]. By contrast, MC-3.5, MC-4.15, MLC-3.5 and MLC-4.15 presented higher volatile acidity and lower alcohol content than the LC group. This might be caused by the increased pH value due to the process of malolactic fermentation.

### 3.2. Analysis of CIELab Parameters of Cherry Wines

The chromatic characteristics of cherry wine with different organic acid treatments are shown in Table 4. CIELab is a color space that was defined by the International Commission on Illumination in 1976, which includes lightness ($L^{*}$), whose values range from 0 (black) to 100 (white), $a^{*}$ (redness and greenness) and $b^{*}$ (yellowness and blueness) on the hue circle. Moreover, the CIELab parameters of $C_{ab}^{*}$, $h_{ab}^{*}$ and ${\Delta E}^{*}$ were calculated from $a^{*}$ and $b^{*}$, in which $C_{ab}^{*}$ represents the saturation or intensity of color, $h_{ab}^{*}$ refers to the relative amounts of redness and yellowness, and ${\Delta E}^{*}$ stands for the total color difference between the sample and the control [37].

In this study, the $L^{*}$, $b^{*}$ and $C_{ab}^{*}$ values of wines were significantly influenced by organic acids, while the $a^{*}$ and $h_{ab}^{*}$ values were not changed. All groups showed an increase in $b^{*}$ and $C_{ab}^{*}$, which is consistent with the results provided by Sun et al. [4]. The $L^{*}$ values of the malic acid and malic–lactic acid treatment groups varied from 97.35 to 98.47, and there was a general tendency of $L^{*}$ to increase in relation to increased acid addition, whereas high lactic acid content presented the opposite trend. Similarly, the values of ${\Delta E}^{*}$ also increased in these two groups, and the overall trend was proportional to the acid addition. However, MLC-5.45 had the highest ${\Delta E}^{*}$ value, which means the color difference between this group and the control group was the most obvious. The results indicated that the addition of organic acids has a positive effect on the yellow/blue color of cherry wine. However, the effects on color difference were not significant, because the values of ${\Delta E}^{*}$ in most samples were less than one [38]. The color characteristics of fruit wine might be caused by the varying expression of anthocyanins at different pH values. Previous studies suggested that the anthocyanins are present as cis-chalcone or trans-chalcone through tautomerization and isomerization reactions under pH 3–6, featuring a yellow hue [39]. Moreover, copigmentation was recognized as an important factor affecting the color of wine during aging [40], and decreasing the pH value of the media would accelerate this process, enhancing the color of fruit wine [41].

### 3.3. Total Phenolics, Total Flavonoids and Antioxidant Activity

The total phenolics, total flavonoids and antioxidant activity of cherry wines are presented in Table 5. The phenolic and flavonoid contents of wine samples ranged from 528.56 to 606.22 μg/mL and 30.15 to 44.45 μg/mL, respectively. These results are consistent with previous results reported by Xiao et al. [30]. As expected, the total phenolics and flavonoids were significantly increased by adding organic acids. This may be related to the high production of ethanol, which contributes to the dissolution of polyphenols from cherry purees [42]. However, the levels presented earlier increased and later decreased with the increase in organic acid application. And the highest total phenolic and flavonoid contents were observed in MC-3.50 and MLC-4.15, in which they increased by 10.04%, 13.44% and 6.98%, 30.74%, respectively. Similarly, the trend of antioxidant activity was the same as that of the total phenolics and flavonoids in different treatment groups. However, MLC-4.15 and LC-3.50 had higher antioxidant activity, which increased by 9.91% and 9.70%, respectively. Previous research suggested that phenolic compounds are potential copigments which can combine with anthocyanin via a π-π stacking interaction [43]. While the copigmentation reactions are strongly affected by pH, the copigmentation concentrations, the solvents and molecule structures, and the losses of polyphenols were possibly caused by the change in environment during wine production [41].

### 3.4. UPLC-Q-TOF/MS Analysis

**Figure 2:**
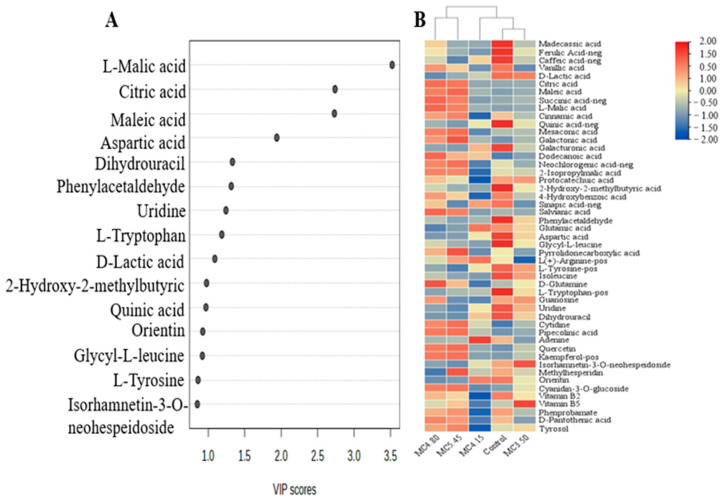
OPLS-DA results (**A**) and heatmap (**B**) of wine samples obtained by adjusting initial acid content (3.50, 4.15, 4.80 and 5.45 g/kg, respectively) of cherry must with malic acid.

**Figure 3:**
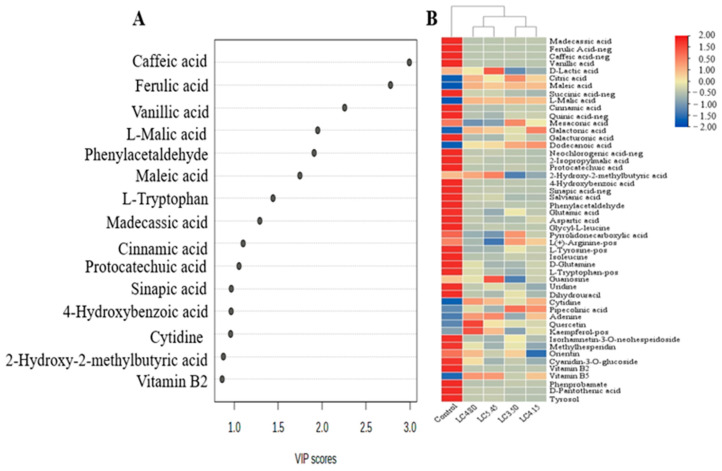
OPLS-DA results (**A**) and heatmap (**B**) of wine samples obtained by adjusting initial acid contents (3.50, 4.15, 4.80 and 5.45 g/kg, respectively) of cherry must with lactic acid.

**Figure 4:**
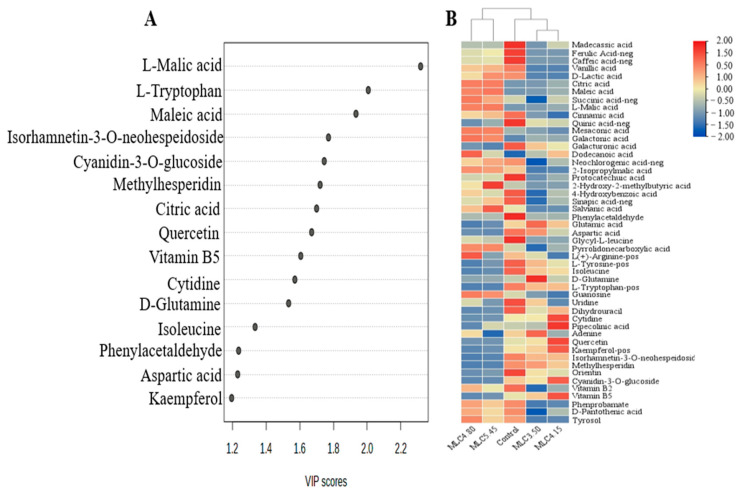
OPLS-DA results (**A**) and heatmap (**B**) of wine samples obtained by adjusting the initial acid contents (3.50, 4.15, 4.80 and 5.45 g/kg, respectively) of cherry must with the combination of malic acid and lactic acid.

Non-volatile compound content in wine samples was analyzed by using UPLC-Q-TOF/MS. A total of 49 metabolites were identified (listed in Table S1), including 22 organic acids, 10 amino acids and derivatives, 6 nitrogen glycosides, 5 flavones, 1 anthocyanidin and 5 others. The relative concentrations of non-volatile compound contents in different groups are illustrated using the heatmap, and the main differential compounds are represented by the VIP score loading plot (Figure 2, Figure 3 and Figure 4).

The application of malic acid before fermentation produced different effects on 4 types of compounds, including 4 organic acids (malic acid, citric acid, maleic acid and lactic), 2 amino acids and derivatives (L-tryptophan and aspartic acid), 2 nitrogen glycoside (uridine and dihydrouracil) and 1 other (phenylacetaldehyde) (Figure 2A). Compared with the control, the contents of malic acid in cherry wine did not change significantly when the initial acid content was no more than 4.15 g/kg, while it increased significantly after the initial acid content adjusting to 4.80 g/kg (Figure 2B). It indicated that the utilization of malic acid was less than its addition in MC-4.80, and resulting in the accumulation of malic acid. In addition, it was found that the content of citric acid and maleic acid also increased, while the content of lactic acid decreased. However, the consumption of amino acids and nitrogen glycoside were increased as the increasing of malic acid addition (Figure 2B). It indicated that the organic acid metabolism was affected by excessive malic acid addition, but had no effect on strain activity during fermentation. Instead of that, the utilization of nitrogen source was promoted by higher malic acid addition.

As for the lactic acid treatment groups, 10 main differentiation compounds were observed according OPLS-DA analysis, which included 8 organic acids, 1 amino acid and 1 other (Figure 3A). It can be seen from Figure 3B that the concentrations of most organic acids were reduced in LC-3.50, but there were no significant effects on their metabolism after increased lactic acid addition. Most interestingly, the addition of lactic acid before fermentation significantly reduced the consumption of malic acid in cherry must, and the lactic acid content of cherry wine did not increase but decreased in the LC-3.50, LC-4.15 and LC-4.80 groups; a significant accumulation of lactic acid was found in LC-5.45. This indicated that lactic acid could be utilized during cherry wine fermentation, and its addition was excessive in LC-5.45.

In the MLC group, more than 15 compounds had high VIP scores, which meant it was considered the most metabolically active group. Similar to the malic acid and lactic acid treatment groups, their combined treatment had significant effects on organic acid, amino acid and nitrogen glycoside metabolism (Figure 4A). According to Figure 4B, the metabolism of malic acid in the MLC group was the same as that in the MC group, and its accumulation could also be observed when the initial acid content was 4.80 g/kg. The difference is that the lactic acid content in the MLC group showed a downward trend until the initial acid concentration was adjusted to 5.45 g/kg, and then returned to the same level as the control. Conversely, the concentrations of amino acids (L-tryptophan, D-glutamine and aspartic acid) and nitrogen glycoside (cytidine) decreased significantly in MLC-5.45. Apart from this, lower concentrations of quercetin, kaempferol, isorhamnetin-3-O-neohespeidoside and cyanidin-3-O-glucoside were found in MLC-4.80 and MLC-5.45. This demonstrated that there was no positive effect on the metabolism of flavones and anthocyanins when the initial acidity was adjusted to more than 4.15 g/kg, and this result was consistent with the analysis results for total flavonoids.

### 3.5. E-Nose Analysis

**Figure 5:**
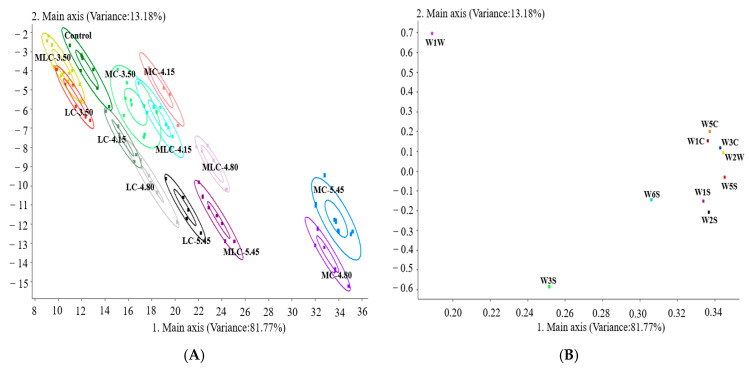
E-nose data on control and cherry wine with different organic acids. (**A**) Score plot; (**B**) loading plot.

The E-nose employs specific sensors and pattern recognition systems to quickly provide comprehensive aroma information on samples [44]. Based on the data obtained from the E-nose (Table S2), the information in Figure 5 was obtained using the principal component analysis method (PCA), which facilitates a more intuitive representation of the aroma disparities among different cherry wines. It can be seen that this model elucidated 94.95% of the total contribution rate (PC1 was 81.77%, PC2 was 13.18%), providing a good explanation of the aroma distinctions among the cherry wine samples. Not all cherry wine samples were clearly separated in PC1 and PC2, which indicated that the aroma components of some cherry wines were similar. As shown in Figure 5A, the locations of cherry wines with low organic acid addition were close to the control, and there was overlap among the different organic acid treatment groups, while significant distinctions were observed with the increase in organic acid addition. This indicated that similar aroma profiles were present in the different treatment groups, while the effects of the different organic acids were various, resulting in significant differences among them with the aroma enhancement of acids.

Notably, all samples exhibited elevated response values in the W5S, W2S, W1S and W2W responders, indicating an abundance of nitrogen oxides, broad-alcohols and short-chain aromatic and sulfide compounds. However, the various cherry wines displayed distinct response values for these sensors. In comparison with the control, the response values for W2S in the treatment groups were significantly increased, indicating that the addition of acids enhanced the synthesis or release of alcohols and aldehydes, while the W5S response value only increased in the LC group, but had no significant difference in the MC and MLC groups. This means the addition of lactic acid probably enhanced the nitrogen oxide production. In addition, higher W1C and W5C values were detected in the MC group; both sensors were sensitive to aromatic compounds. The results suggest that the E-nose could properly characterize the cherry wine samples, but the specific differences among these samples needed to be further identified by GC-MS due to the limitations of E-nose analysis.

### 3.6. GC-MS Analysis

**Figure 6:**
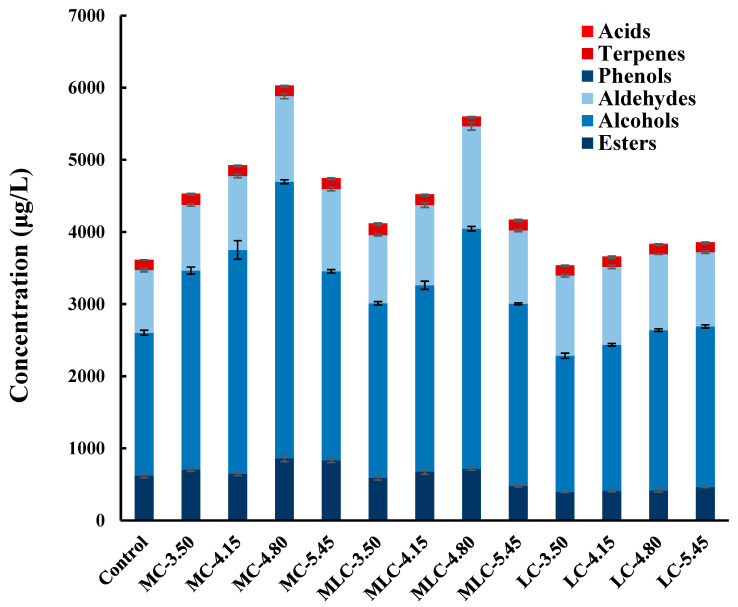
Volatile organic compound concentration histogram of control and different organic acid treatment groups.

**Figure 7:**
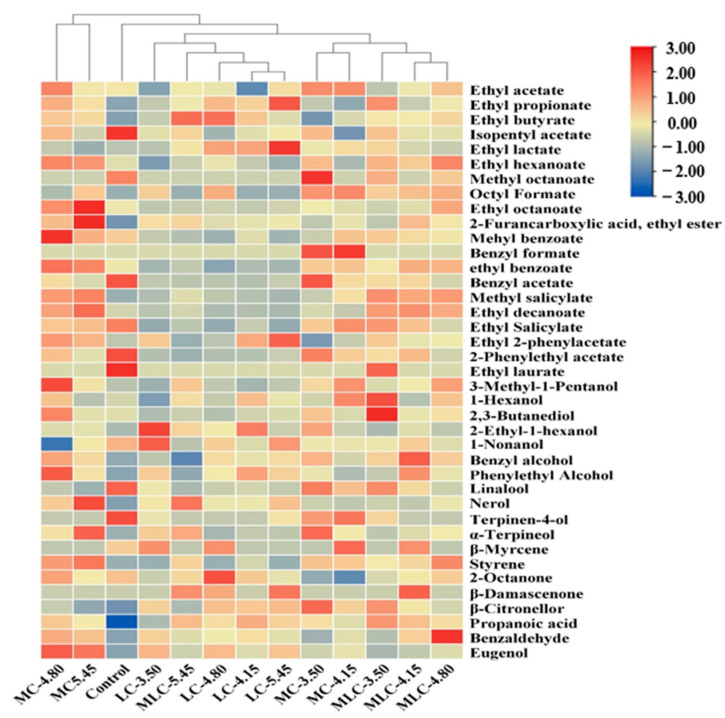
Heatmap visualization of relative differences of volatile organic compounds in cherry wines. The color indicates the level of accumulation of each metabolite, from low (blue) to high (red).

A total of 39 volatile compounds were identified and quantified by using headspace (HS)-SPME/GC-MS, including 20 esters, 7 alcohols, 9 terpenes, 1 acid, 1 aldehyde and 1 phenol (Table S3). The concentration histogram of various kinds of VOCs in different cherry wines is presented in Figure 6, and the metabolic differences in VOCs in cherry wines treated by different organic acids were identified using a heatmap (Figure 7).

As can be seen in Figure 6, the addition of organic acids had positive effects on the synthesis and release of volatiles. The total contents of VOCs were increased significantly in all treatment groups, among which MC-4.80, MLC-4.80 and LC-5.45 showed the highest enhancements of total VOCs, which increased by 66.92%, 55.04% and 6.83%, respectively. Alcohols and aldehyde were the most abundant VOCs in cherry wine, accounting for 54.88% and 24.08% of the total VOCs in the control. The addition of malic acid and malic–lactic acid significantly affected the metabolism of these two kinds of compounds, and was promoted by the increase in organic acids. Among them, MC-4.80 and MLC-4.80 increased by 93.36%, 36.50% and 67.79%, 63.14%, respectively. Similarly, the addition of lactic acid could also improve the alcohol and aldehyde contents in cherry wine, while the correlation with the usage was not obvious. Moreover, the most interesting observation is that the content of esters increased significantly after adding malic acid and malic–lactic acid, while it decreased in the lactic acid group. Due to the different compositions and concentrations of VOCs in different cherry wines, a hierarchical cluster analysis is presented in Figure 7.

Alcohols were the largest group of volatiles in all samples. 3-methyl-1-pentanol and benzyl alcohol were the dominant alcohols, accounting for 54.70% and 41.66% in the control group (Table S4), which is in agreement with the results reported by Sun et al. [45]. The concentrations of these two compounds increased significantly when organic acids were used, and the largest increase in 3-methyl-pentanol was detected in MC-4.80, as high as 139.76%, while the cherry wine fermented with malic–lactic acid was distinguished by a higher amount of benzyl alcohol, which increased by 49.94% in MLC-4.80, and which is associated with floral and sweet notes [46]. By contrast, the use of lactic acid resulted in a significantly lower production of these two compounds, which increased by 3.94% and 22.30% in LC-4.80. Moreover, phenylethyl alcohol, which contributes a sweet and rosy flavor to the wine [47], also evidently increased among the tested samples, and the highest concentrations were observed in MC-4.80 (98.66 μg/L). This indicated that the addition of different organic acids could enhance the concentration of alcohols, while it has no effect on the composition of alcohols. Previous research suggested that alcohols are mainly formed by the deamination and decarboxylation reactions from amino acids [15,48], and the low pH level of alcoholic fermentation favors the development of more alcohols [33].

Esters are mainly formed from esterification of alcohols with fatty acids during the fermentation and aging process, and are characterized by a fruity and sweet flavor, which significantly contributes to the construction of the characteristic cherry wine aroma [4,49]. Among the esters, ethyl benzoate, ethyl acetate and ethyl octanoate were the main esters in the control, accounting for 47.91%, 15.28% and 8.75%, respectively (Table S4). Malic acid and its combination with lactic acid positively influenced the synthesis of ethyl benzoate and ethyl acetate in cherry wine. The highest concentration of ethyl benzoate was present in MC-4.80 (464.60 μg/L), as was also the case for ethyl acetate (125.01 μg/L). Similarly, the same trend was observed in ethyl octanoate, which increased by 184.58% (MC-5.45) and 72.11% (MLC-4.80). However, the addition of lactic acid did not promote their formation significantly, and even reduced the levels of ethyl benzoate and ethyl octanoate. In addition, decreases in isopentyl acetate and benzyl acetate were noted in all the acid addition groups. Bartowsky et al. [36] suggest that the content of esters could rise or decline in the process of malolactic fermentation. And this is associated with esterase activity, malic acid consumption, pH value and ethanol content [15,45]. Moreover, it is interesting to note that the LC group showed the highest ethyl lactate content among all the organic acid treatment groups. The content of ethyl lactate was significantly proportional to the lactic acid addition, which means that the consumed lactic acid might be directly transformed into ethyl lactate by esterification during wine aging [22].

Terpenes are mainly released from their glycoside during the alcoholic fermentation stage, and contribute to the wine with fruity, floral or spicy notes [50,51]. In the current study, the kinds of volatiles were not affected by the addition of acids, while the compositions varied obviously. The level of linalool decreased significantly with the addition of organic acids, decreasing by 7.09–53.62% in the MC group, 7.09–53.62% in the MLC group and 33.29–45.24% in the LC group when the initial acids were adjusted from 3.50 to 5.45 g/kg. On the other hand, the α-terpineol and β-citronellol contents in all the organic acid treatment groups increased significantly, and the highest levels were detected in the treatment group with the highest acid content (5.45 g/kg). Knoll et al. [15] and Sun et al. [45] suggested that the abilities of lactic acid bacteria to hydrolyze and release glucoside-bound aromatic compounds may not be linked to the metabolism of malolactic fermentation, and may only be dependent on the bacterial strain. This indicated that the addition of acids to enhance malolactic fermentation may have no effect on the release of terpenes. But the decrease in pH caused by this leads to the rearrangements of linalool into α-terpineol, β-citronellol and nerol [45,52], which contribute significantly to the varietal flavor and aroma complexity of cherry wine.

Benzaldehyde, commonly known for its almond and burnt sugar notes, was the only aldehyde compound detected in cherry wine. A similar result was reported by Sun et al. [4]. During this study, the levels of benzaldehyde were significantly increased from 909.56 to 1187.53 μg/L in the MC group and from 946.10 to 1419.32 μg/L in the MLC group when the initial acid increased from 3.50 to 4.15 g/kg. After that, there was no positive effect on the formation of benzaldehyde with increases in the initial acid concentration. It is interesting that LC-3.50 showed the highest concentration of benzaldehyde in the LC group. This means that lactic acid might be more efficient in the formation of benzaldehyde. Sun et al. [45] and Genovese et al. [47] suggested that benzaldehyde is mainly formed by the oxidation of benzyl alcohol or the action of microorganisms on aromatic amino acids during malolactic fermentation, and the difference among samples is caused by the utilization of organic acids during malolactic fermentation. Furthermore, small amounts of propanoic acid and eugenol were detected in some samples, imparting cherry wine with a soy and flowery aroma [26,46,53].

### 3.7. Sensory Evaluation

**Figure 8:**
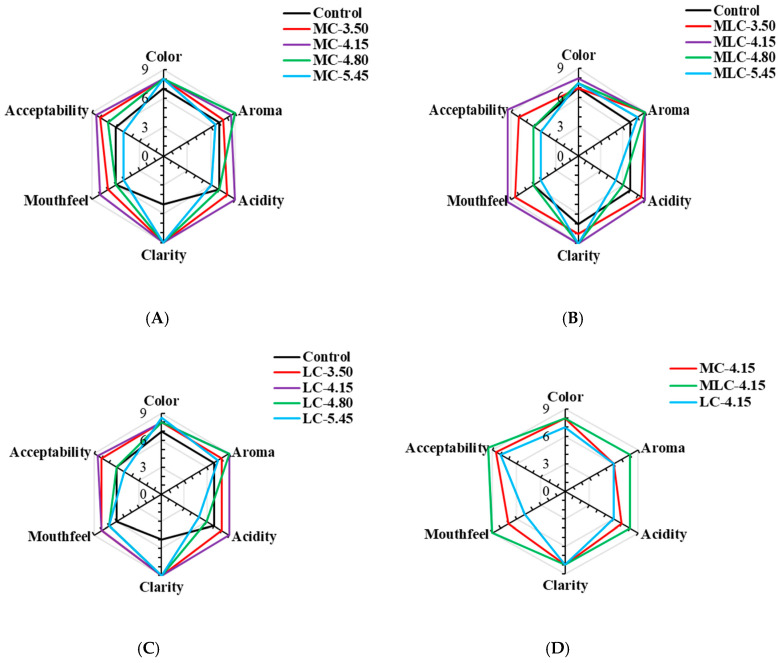
Sensory evaluation radar chart of cherry wine samples obtained by adjusting the initial acid content (3.50, 4.15, 4.80 and 5.45 g/kg, respectively) of cherry must with different organic acids. (**A**) Sensory attributes of cherry wine adjusted with malic acid. (**B**) Sensory attributes of cherry wine adjusted with the mixture of malic acid and lactic acid. (**C**) Sensory attributes of cherry wine adjusted with lactic acid. (**D**) Sensory evaluation of cherry wine samples with the highest score in each group.

The sensory evaluation of the color, aroma, acidity, clarity, mouthfeel and acceptability of cherry wine samples fermented with different kinds of organic acid additions is illustrated in Figure 8. The results indicated that organic acid addition had a significant effect on all of the sensory attributes (*p* < 0.05). Specifically, the satisfaction with the clarity of the cherry wine was significantly higher than the control (*p* < 0.05). Moreover, the increasing addition of organic acids had significant effects on aroma, acidity, mouthfeel and acceptability in each treatment group, while there was no significant difference in the effects on color and clarity, except in the MLC group (*p* < 0.05). In each group, cherry wine obtained by adjusting the initial acid content to 4.15 and 4.80 g/kg had the highest aroma satisfaction, which means these two initial acid concentrations had a positive effect on the aroma release. However, the acidity satisfaction with cherry wine was reduced when the initial acidity content was 4.80 g/kg, as was the mouthfeel satisfaction, which resulted in lower acceptability of cherry wine.

Thus, the contributions of different organic acids (the initial acid content was 4.15 g/kg) to the sensory properties of cherry wines were compared in this study (Figure 8D). It indicated that the addition of different organic acids had significant effects on the aroma, acidity and mouthfeel of cherry wine, while the contribution to the clarity of wines was not obvious. Among the samples, MLC-4.15 scored the highest in aroma, acidity, mouthfeel and acceptability, which means it is more likely to be chosen by customers.

## 4. Conclusions

In this study, the effects of organic acid addition before fermentation on the quality of cherry wine were explored. The results suggest that the addition of organic acid effectively reduced the volatile acid content to the acceptable limit, and increased the ethanol content as well. Both malic acid and lactic acid could be used as carbon sources during cherry wine fermentation, and their excessive addition occurred in the MC-4.80, LC-5.45 and MLC-4.80 groups. In the MC and MLC groups, the metabolism of non-volatile compounds was obviously promoted when the initial acidity was adjusted to no more than 4.15 g/kg, and the highest VOC content was observed when the initial acid content was 4.80 g/kg. Lactic acid had little effect on VOCs in cherry wine, except on ethyl lactate. The contents of 3-methyl-1-pentanol, benzyl alcohol and benzaldehyde were significantly increased during fermentation, which enhanced the floral, sweet and almond flavors of cherry wine. However, MLC-4.15 showed the highest overall acceptability in sensory evaluation due to its sourness property and balanced mouthfeel. Thus, it is recommended that the initial acid content be adjusted to 4.15 g/kg by using the mixture of malic acid and lactic acid for the production of dry cherry wine (residual sugar ≤ 4 g/L). If cherry wine is used to produce semi-dry (residual sugar ≤ 12 g/L) or semi-sweet wine (residual sugar ≤ 45 g/L), it is recommended that the initial acidity be adjusted to 4.80 g/kg because of its high aroma content.

## Figures and Tables

**Table 1:** The mobile phase and gradient profile from the UPLC-Q-TOF analysis.

Time (min)	Flow Rate (mL/min)	Mobile Phase A (Acetonitrile)	Mobile Phase B (0.1% Formic Acid Aqueous Solution, *v*/*v*)
0	0.25	5	95
3	0.25	10	90
12	0.25	30	70
18	0.25	60	40
19	0.25	80	20
25	0.25	100	0
27	0.25	100	0
28	0.25	5	95
30	0.25	5	95

**Table 2:** The sensors and their main properties in the PEN3 electronic nose.

Sensor	Responsive Substance	Performance Description	Threshold Value
W1C	Aromatics	Sensitive to aromatic compounds	10 ppm
W5S	Broad range	Sensitive to nitrogen oxides	NO_2_, 1 ppm
W3C	Aromatic	Sensitive to ammonia, aromatic compounds	Benzene, 10 ppm
W6S	Hydrogen	Sensitive to hydride	H_2_, 100 ppb
W5C	Arom–aliph	Sensitive to short-chain alkanes, aromatic compounds	Propane, 1 ppb
W1S	Broad–aliph	Sensitive to short-chain alkanes, aromatic compounds	CH_3_, 100 ppm
W1W	Sulfur–organic	Sensitive to sulfides, pyrazine and many terpenes	H_2_S, 1 ppm
W2S	Broad–alcohol	Sensitive to alcohols, aldehydes and ketones	CO, 100 ppm
W2W	Sulf–chlor	Sensitive to aromatics, sulfur and chlorine containing organics	H_2_S, 1 ppm
W3S	Methane–aliph	Sensitive to methane and alkanes	CH_3_, 100 ppm

**Table 3:** The oven temperature program for GC-MS analysis.

Temperature Gradient	Temperature Rate (°C/min)	Temperature (°C)	Maintained Time (min)	Total Run Time (min)
Initial		33	1	1
1	3	78	2	18
2	3	90	2	24
3	3	99	2	29
4	3	140	1	43.667
5	6	160	1	48
6	8	220	2	57.9

**Table 4:** Comparison and evolution of the CIELab parameters ($L^{*},\;a^{*},\;b^{*},\;C_{ab}^{*},\;h_{ab}\;and\;{\Delta E}^{*}$) of cherry wine with different organic acid treatments.

Sample	$L^{*}$	$a^{*}$	$b^{*}$	$C_{ab}^{*}$	$h_{ab}$	${\Delta E}^{*}$
Control	97.99 ± 0.06 ^d^	0.84 ± 0.72 ^a^	4.75 ± 0.59 ^d^	4.87 ± 0.42 ^b^	1.39 ± 0.18 ^b^	-
MC-3.50	97.98 ± 0.10 ^d^	0.53 ± 0.08 ^ab^	4.81 ± 0.29 ^bc^	4.84 ± 0.28 ^b^	1.46 ± 0.02 ^ab^	0.40 ± 0.07 ^d^
MC-4.15	98.09 ± 0.06 ^cd^	0.51 ± 0.10 ^ab^	4.84 ± 0.21 ^bcd^	4.86 ± 0.20 ^b^	1.47 ± 0.02 ^ab^	0.40 ± 0.12 ^d^
MC-4.80	98.37 ± 0.04 ^ab^	0.58 ± 0.15 ^ab^	4.71 ± 0.39 ^d^	4.75 ± 0.38 ^b^	1.45 ± 0.04 ^ab^	0.57 ± 0.12 ^bcd^
MC-5.45	98.42 ± 0.04 ^ab^	0.61 ± 0.14 ^ab^	5.27 ± 0.07 ^ab^	5.31 ± 0.08 ^a^	1.46 ± 0.02 ^ab^	0.72 ± 0.08 ^bc^
MLC-3.50	97.35 ± 0.25 ^e^	0.37 ± 0.11 ^b^	4.82 ± 0.06 ^bcd^	4.84 ± 0.06 ^b^	1.50 ± 0.02 ^a^	0.80 ± 0.26 ^ab^
MLC-4.15	98.23 ± 0.25 ^bc^	0.45 ± 0.08 ^ab^	4.84 ± 0.06 ^bcd^	4.86 ± 0.06 ^b^	1.48 ± 0.02 ^ab^	0.51 ± 0.14 ^cd^
MLC-4.80	98.14 ± 0.04 ^cd^	0.54 ± 0.07 ^ab^	5.28 ± 0.08 ^ab^	5.31 ± 0.07 ^a^	1.47 ± 0.02 ^ab^	0.62 ± 0.09 ^bcd^
MLC-5.45	98.47 ± 0.01 ^a^	0.56 ± 0.02 ^ab^	5.59 ± 0.07 ^a^	5.62 ± 0.06 ^a^	1.47 ± 0.00 ^ab^	1.00 ± 0.06 ^a^
LC-3.50	98.46 ± 0.07 ^a^	0.61 ± 0.01 ^ab^	4.27 ± 0.17 ^e^	4.31 ± 0.16 ^c^	1.43 ± 0.01 ^ab^	0.72 ± 0.15 ^bc^
LC-4.15	98.03 ± 0.10 ^cd^	0.46 ± 0.04 ^ab^	5.36 ± 0.21 ^a^	5.38 ± 0.20 ^a^	1.49 ± 0.01 ^ab^	0.73 ± 0.20 ^bc^
LC-4.80	98.02 ± 0.02 ^d^	0.50 ± 0.01 ^ab^	5.24 ± 0.12 ^abc^	5.26 ± 0.11 ^a^	1.48 ± 0.00 ^ab^	0.60 ± 0.10 ^bcd^
LC-5.45	97.93 ± 0.04 ^d^	0.49 ± 0.04 ^ab^	5.42 ± 0.14 ^a^	5.44 ± 0.13 ^a^	1.48 ± 0.01 ^ab^	0.76 ± 0.14 ^bc^

Note: Different lowercase letters in the same column represent significant differences (ANOVA, *p* < 0.05). (MC: malic acid; MLC: malic acid and lactic acid; LC: lactic acid; ANOVA: analysis of variance).

**Table 5:** Total phenolic content, total flavonoid content and antioxidant activity of cherry wine with different organic acid treatments.

Sample	Total Phenolic Content (μg GAE/mL)	Total Flavonoid Content (μg CE/mL)	DPPH Scavenging Activity (%)
Control	550.90 ± 2.92 ^f^	34.00 ± 0.30 ^de^	52.70 ± 0.26 ^bc^
MC-3.50	606.22 ± 0.17 ^a^	38.57 ± 0.10 ^c^	53.14 ± 0.49 ^bc^
MC-4.15	567.56 ± 1.03 ^e^	34.28 ± 0.67 ^de^	50.78 ± 0.40 ^cde^
MC-4.80	578.56 ± 2.75 ^cd^	34.10 ± 2.05 ^de^	46.90 ± 1.11 ^f^
MC-5.45	577.35 ± 0.86 ^d^	30.23 ± 2.03 ^f^	47.32 ± 0.94 ^f^
MLC-3.50	574.26 ± 3.95 ^d^	35.55 ± 0.28 ^d^	48.97 ± 1.12 ^def^
MLC-4.15	589.38 ± 0.17 ^b^	44.45 ± 3.35 ^a^	57.92 ± 4.07 ^a^
MLC-4.8	582.68 ± 5.84 ^c^	31.45 ± 0.25 ^ef^	46.81 ± 0.69 ^f^
MLC-5.45	528.56 ± 2.92 ^g^	30.15 ± 0.08 ^f^	48.06 ± 3.42 ^ef^
LC-3.50	591.79 ± 0.86 ^b^	42.16 ± 1.45 ^ab^	57.83 ± 0.32 ^a^
LC-4.15	589.90 ± 4.47 ^b^	41.10 ± 3.90 ^bc^	55.36 ± 1.23 ^ab^
LC-4.80	577.87 ± 1.03 ^cd^	34.41 ± 0.31 ^de^	53.10 ± 0.16 ^bc^
LC-5.45	552.27 ± 0.52 ^f^	34.28 ± 0.27 ^de^	51.85 ± 1.37 ^cd^

Note: Different lowercase letters in the same column represent significant differences (ANOVA, *p* < 0.05). (MC: malic acid; MLC: malic acid and lactic acid; LC: lactic acid; ANOVA: analysis of variance).

## Data Availability

The original contributions presented in the study are included in the article/Supplementary Material, further inquiries can be directed to the corresponding author.

## Supplementary Materials

The following supporting information can be downloaded at https://www.mdpi.com/article/10.3390/foods13233902/s1. Figure S1. (A): The total ion chromatogram of MLC-4.15 in positive mode (blue line is the total ion chromatogram of MS spectrum, and the red line is the total ion chromatogram of MS/MS); (B): The precursor mass information for IDA (information dependent acquisition). Figure S2. (A): The total ion chromatogram of MLC-4.15 in negative mode (blue line is the total ion chromatogram of MS spectrum, and the red line is the total ion chromatogram of MS/MS); (B): The precursor mass information for IDA (information dependent acquisition). Figure S3. The chromatogram of of LC-4.80. Table S1. Information of 59 identified compounds in cherry wine by using UPLC-Q-TOF-MS. Table S2. Electronic nose (E-nose) response data of control and different organic acids treatment groups at 192 s. Table S3. The concentration of individual volatile organic compounds in control and different organic acids treatment groups (μg/L). Table S4. The contents of different categories volatile organic compounds in control and different organic acids treatment groups (μg/L).
