# Supplementary material for: Effect of Organic Acid Addition Before Fermentation on the Physicochemical and Sensory Properties of Cherry Wine
Source: Foods. 2024 Dec 3;13(23):3902. doi: 10.3390/foods13233902 (PMC11640468; doi:10.3390/foods13233902)
Supplement: Supplementary file 1 [file foods-13-03902-s001.zip › Foods-Supplement material Figures- R2.pdf]

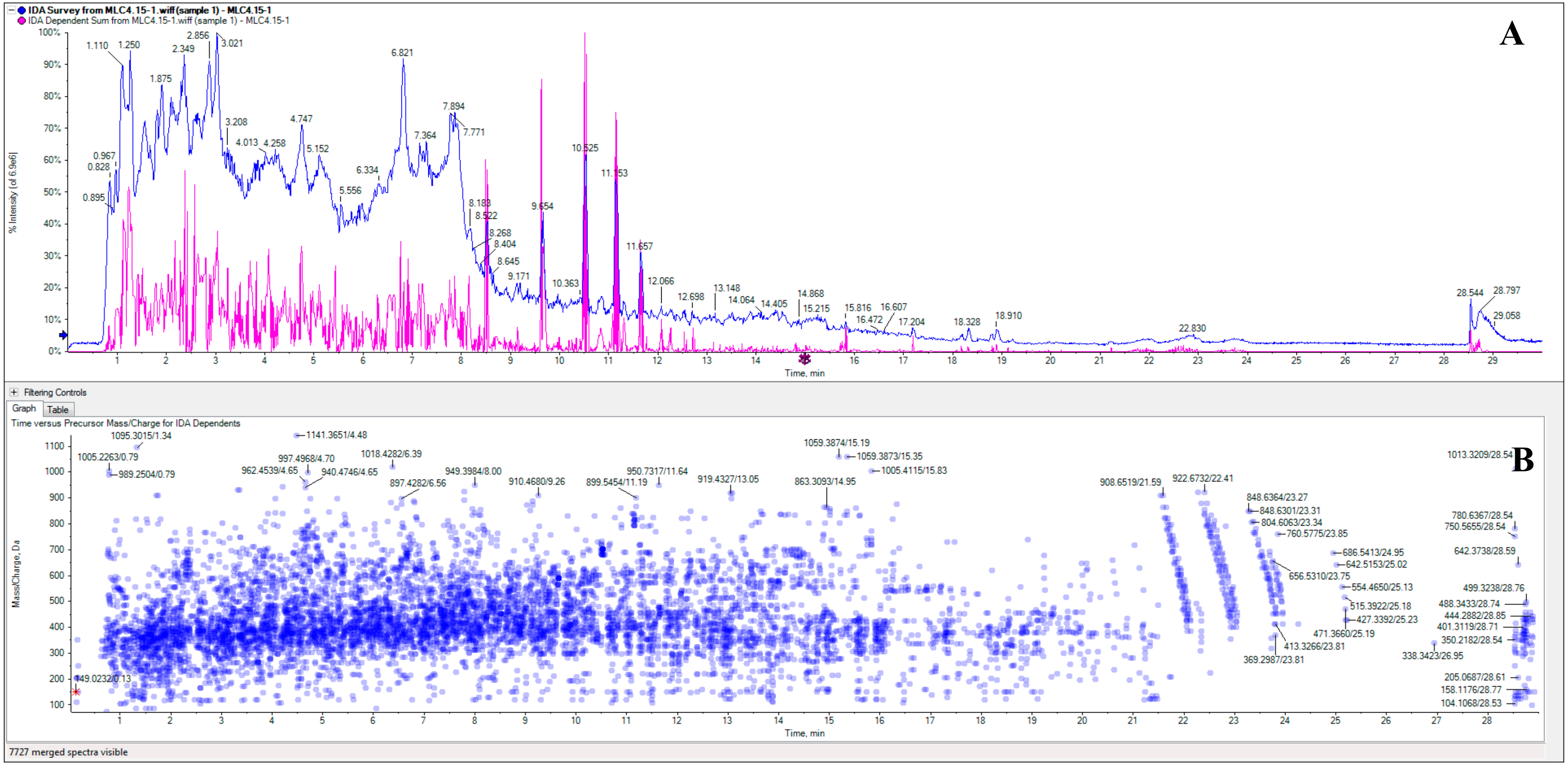


**Figure S1. (A): The total ion chromatogram of MLC-4.15 in positive mode (blue line is the total ion chromatogram of MS spectrum, and the red line is the total ion chromatogram of MS/MS); (B): The precursor mass information for IDA (information dependent acquisition).**

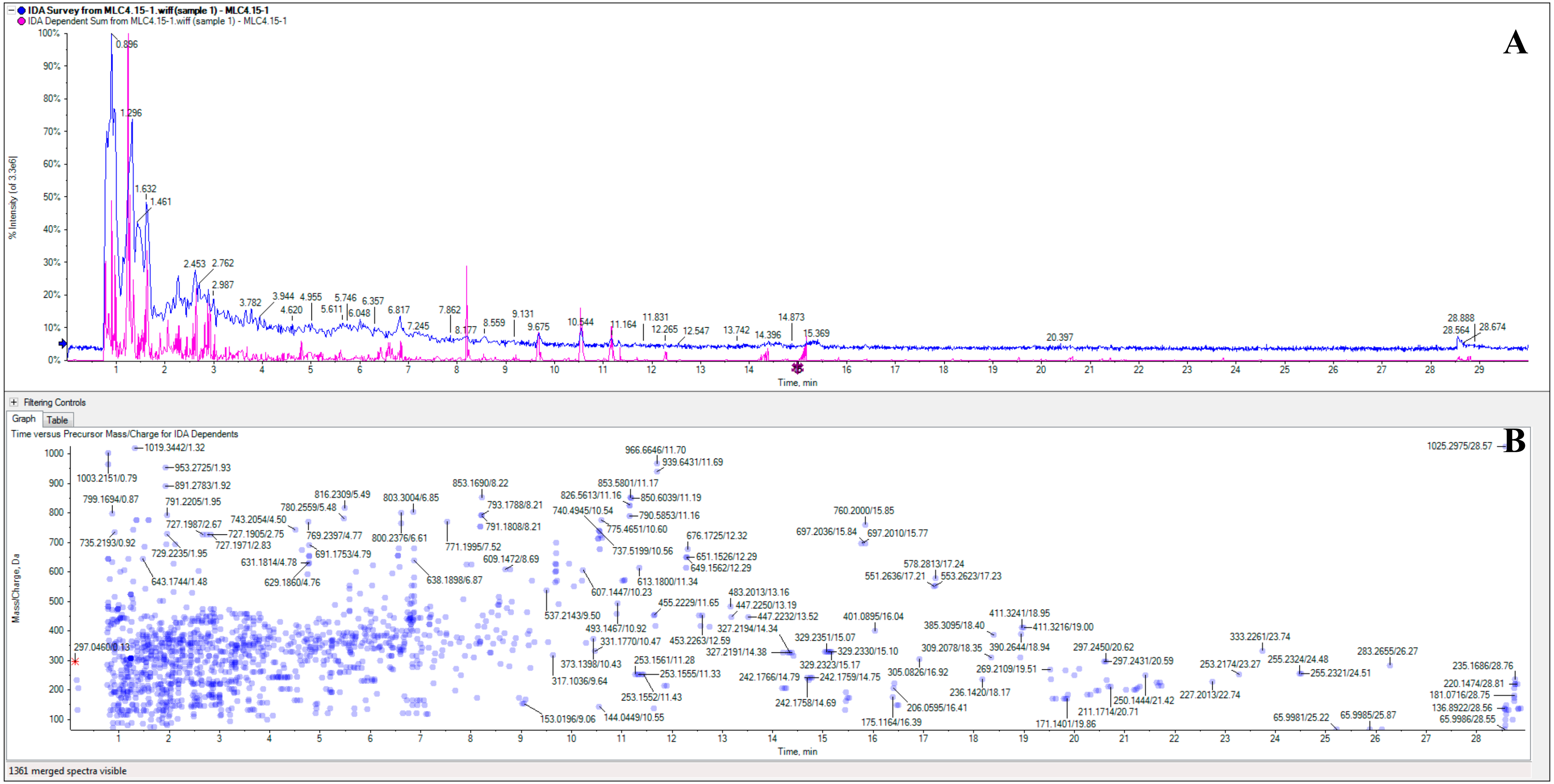

**Figure S2. (A): The total ion chromatogram of MLC-4.15 in negative mode (blue line is the total ion chromatogram of MS spectrum, and the red line is the total ion chromatogram of MS/MS); (B): The precursor mass information for IDA (information dependent acquisition).**

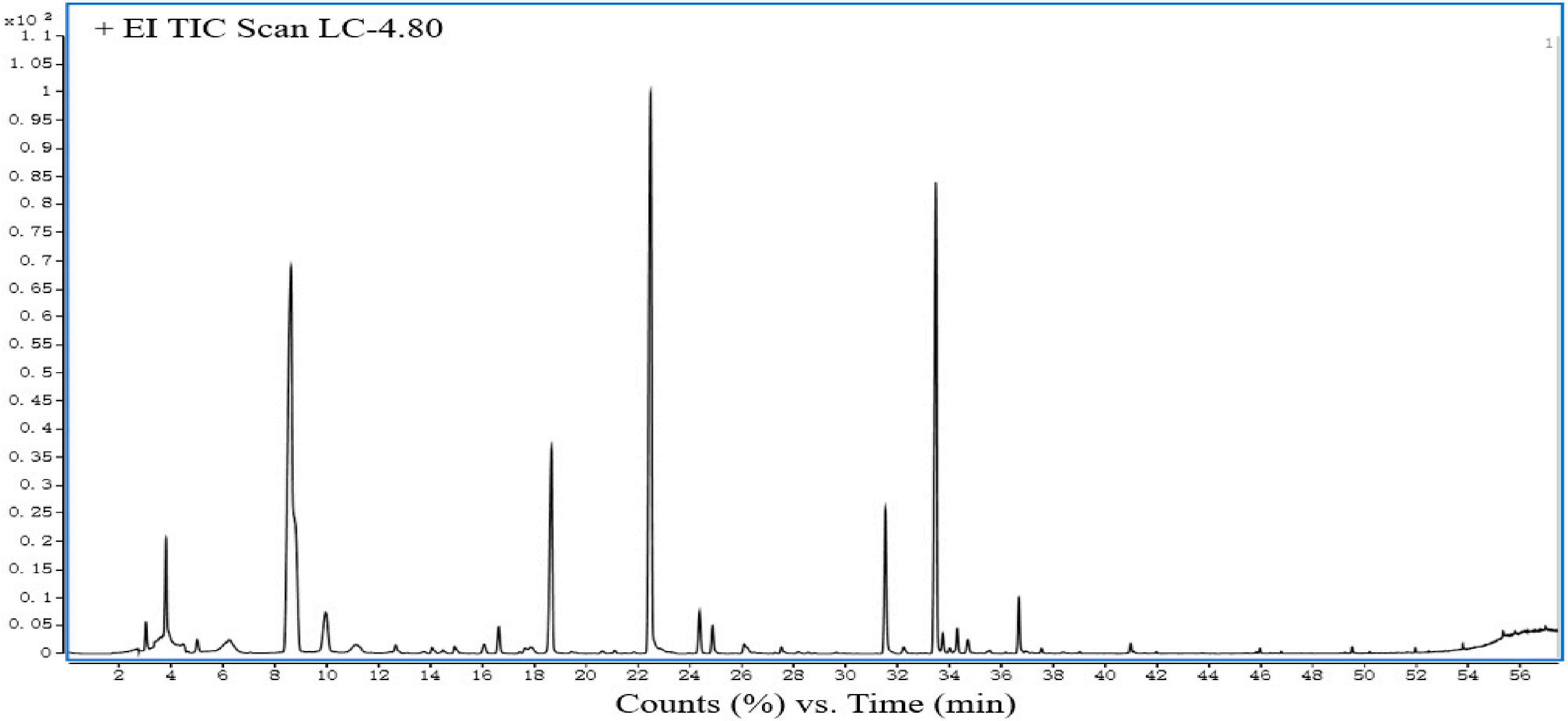


**Figure S3. The chromatogram of of LC-4.80.**
